# Assisted Design of Antibody and Protein Therapeutics (ADAPT)

**DOI:** 10.1371/journal.pone.0181490

**Published:** 2017-07-27

**Authors:** Victor Vivcharuk, Jason Baardsnes, Christophe Deprez, Traian Sulea, Maria Jaramillo, Christopher R. Corbeil, Alaka Mullick, Joanne Magoon, Anne Marcil, Yves Durocher, Maureen D. O’Connor-McCourt, Enrico O. Purisima

**Affiliations:** Human Health Therapeutics, National Research Council Canada, Montreal, QC, Canada; King's College London, UNITED KINGDOM

## Abstract

Effective biologic therapeutics require binding affinities that are fine-tuned to their disease-related molecular target. The ADAPT (Assisted Design of Antibody and Protein Therapeutics) platform aids in the selection of mutants that improve/modulate the affinity of antibodies and other biologics. It uses a consensus z-score from three scoring functions and interleaves computational predictions with experimental validation, significantly enhancing the robustness of the design and selection of mutants. The platform was tested on three antibody Fab-antigen systems that spanned a wide range of initial binding affinities: bH1-VEGF-A (44 nM), bH1-HER2 (3.6 nM) and Herceptin-HER2 (0.058 nM). Novel triple mutants were obtained that exhibited 104-, 46- and 32-fold improvements in binding affinity for each system, respectively. Moreover, for all three antibody-antigen systems over 90% of all the intermediate single and double mutants that were designed and tested showed higher affinities than the parent sequence. The contributions of the individual mutants to the change in binding affinity appear to be roughly additive when combined to form double and triple mutants. The new interactions introduced by the affinity-enhancing mutants included long-range electrostatics as well as short-range nonpolar interactions. This diversity in the types of new interactions formed by the mutants was reflected in SPR kinetics that showed that the enhancements in affinities arose from increasing on-rates, decreasing off-rates or a combination of the two effects, depending on the mutation. ADAPT is a very focused search of sequence space and required only 20–30 mutants for each system to be made and tested to achieve the affinity enhancements mentioned above.

## Introduction

The design of superior biologic therapeutics, including monoclonal antibodies, single-domain antibodies, and engineered proteins, involves optimizing their ability to bind to disease-related molecular targets (antigens). Immunization of animals [1] and phage display methods [2] are the workhorses of antibody discovery and optimization. However, these methods do not always achieve the desired levels of affinity and/or specificity. Computational approaches [3–7] provide a complementary strategy for affinity optimization but predicting successful designs reliably has been challenging. One of the most daunting challenges in antibody sequence optimization is prioritizing the sequence space resulting from the combinatorial explosion when mutating multiple sites in complementarity determining region (CDR) loops. For example, for CDR loops containing 60 amino acids there are about 10^9^ different triple mutants possible. This number increases by a factor of 10^3^ for each additional mutation site. Exploring this space computationally through random virtual mutagenesis or other stochastic search methods explores only a small corner of sequence space with no clear stopping criteria. We sought to develop a more deterministic approach for building a focused set of mutations with a high likelihood of improving binding.

The ADAPT (Assisted Design of Antibody and Protein Therapeutics) platform was developed to aid in the selection of mutants that improve/modulate the affinity of antibodies and other biologics. ADAPT interleaves computational predictions with experimental validation, significantly enhancing the robustness of the design and selection of mutants. We briefly explain the workflow of the method. The first step is an exhaustive single-point virtual mutagenesis along the entire CDR sequence. Each amino acid is mutated in turn to each of 18 amino acids (mutation to Cys and Pro is excluded) and a binding affinity score is calculated relative to the parent sequence. The affinity score is a median-based composite z-score using three scoring functions (see Methods section), reflecting how many standard deviations away from the median a particular score is. From the 50 top-scoring mutants approximately 15 mutants are selected based on amino acid and site diversity and manual visual inspection of the virtual mutants. The Fab fragments for these are then produced and their binding affinities measured by SPR. Typically, around 3–4 sites are confirmed experimentally to be favorable sites for mutations. This early experimental validation is critical because it narrows down the subsequent mutations to combinations of just these sites. Thus, we avoid the combinatorial explosion that can occur in constructing double and higher mutants. The double-mutant candidates in the next round are limited to pairs of validated single mutants–up to about 30 distinct double mutants. For a 60-amino acid CDR, this is much less than the theoretical 1.2 x 10^6^ possible double mutants. Similarly, for the construction of triple mutants we restrict our combinations to those of validated double mutants and validated single mutants. Furthermore, the filtering at the single-mutant stage also increases the robustness of the design of higher order mutants. Multiple-site mutants are constructed using only validated single mutants.

This strategy was tested on three antibody Fab-antigen complexes: bH1-VEGF-A, bH1-HER2 and Herceptin-HER2 [8,9]. We chose these three systems because they covered a wide range of initial affinities for the parent sequence, representing increasing challenges for further affinity maturation. All three had crystal structures for the complex, providing a solid starting point for the virtual mutation simulations. Our measured affinities for these by SPR were 44 nM (bH1-VEGF-A), 3.6 nM (bH1-HER2) and 0.058 nM (Herceptin-HER2).

## Results

**Fig 1:**
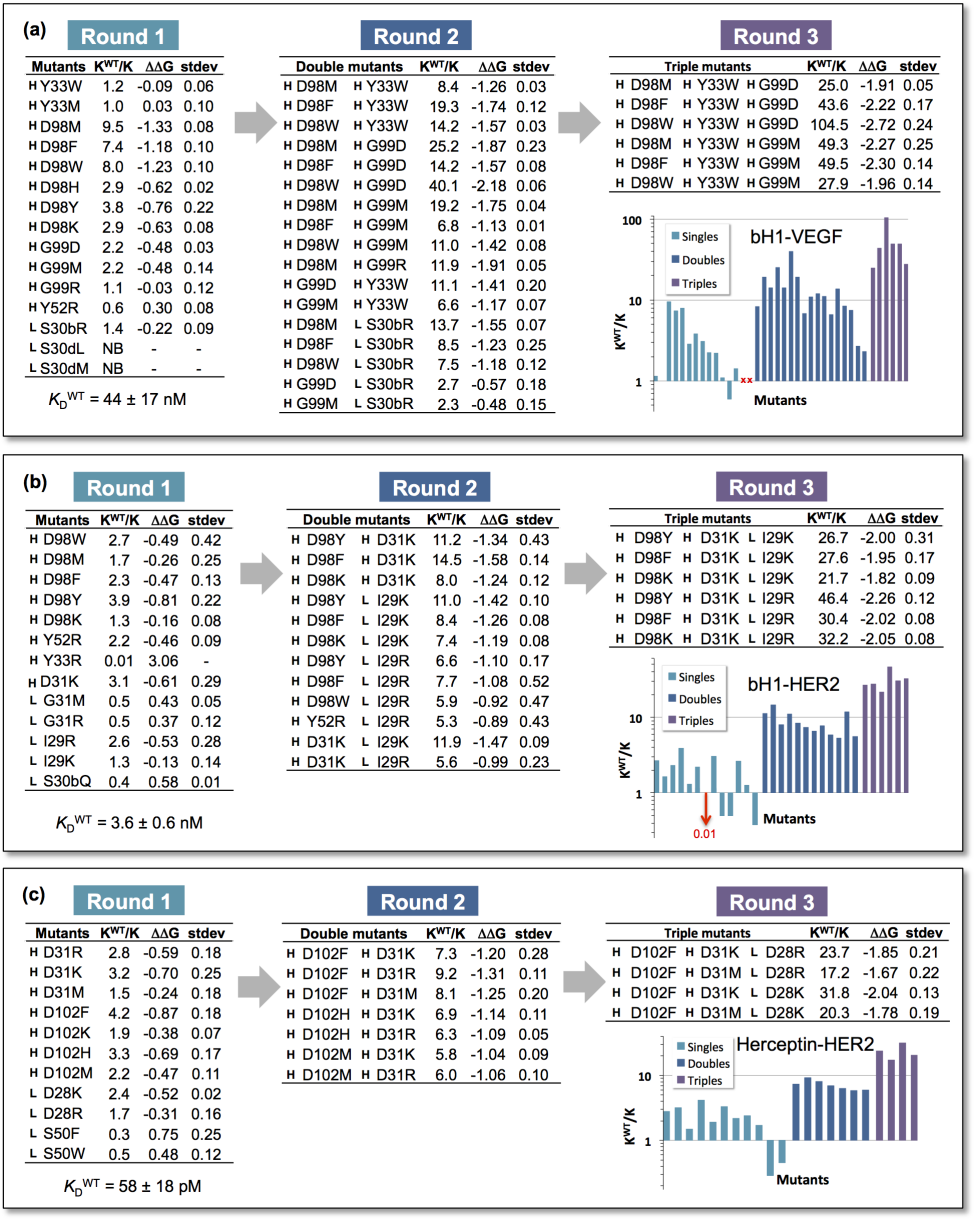
Fold improvements in binding affinity and relative changes in binding free energy (kcal/mol) relative to the parent Fab during three rounds of mutations. (a) bH1-VEGF. (b) bH1-HER2. (c) Herceptin-HER2. H and L designate the mutation as being in the heavy or light chain, respectively. Standard deviations of ΔΔG are based on 3 or more replicates, typically. NB = no binding. WT = parent sequence. Residue numbering for bH1 follows that of Bostrom et al. [8] Residue numbering for Herceptin follows that in the 1n8z PDB entry.

**Fig 2:**
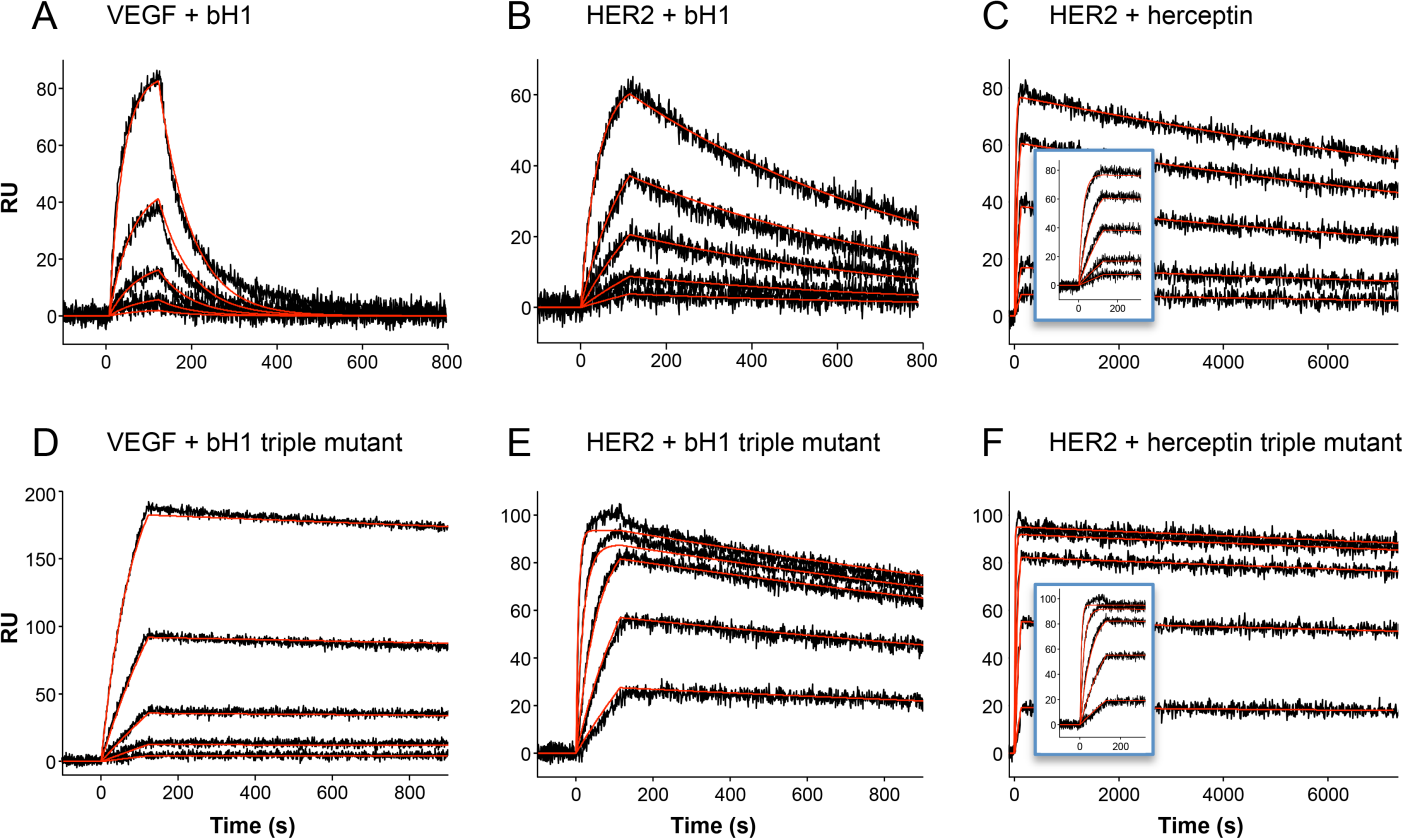
Sensorgrams of the parent Fabs and best triple mutant for each complex. The red curves represent the global fits of the data to a 1:1 bimolecular interaction model. The slow off rates of Herceptin and its triple mutant required longer data acquisition times in the dissociation phase to obtain reliable kinetics. The insets in panels (C) and (F) are expanded views of the association phase of these sensorgrams.

Fig 1A shows the results of three rounds of design for bH1-VEGF. In the first round, 14 diverse single mutants were selected from the top 50 z-scores for experimental validation. The selection was driven not just by the z-scores but also by the desire to sample multiple mutation sites and amino acid types (see Methods section). The selected mutants had z-scores ranging from –8.5 to –1.6 (S1 Table). Of these, 11 mutants showed improvements in affinity ranging from 1.1-fold to 9.5-fold as measured by SPR, two had slightly weaker binding and the remaining two had no detectable binding. The mutants with improved affinity were located at four sites (HY33, HD98, HG99, LS30b) in the CDRs. The designations H and L indicate the residue as being in the heavy or light chain, respectively. For the second round, we tested 17 double mutants selected out of 32 possible ones (S2 Table) arising from combinations of the validated single mutants. All 17 showed improved affinities over the parent ranging from 2.3- to 40-fold, 15 of which showed affinity improvements greater than either of the component single mutants. Given that the LS30bR mutation had the smallest incremental improvement when combined with other single mutations we restricted the third round to triple combinations of heavy chain mutants used in the second round. All six triple mutants that were made showed further improvements in affinity compared to the double mutants (Fig 1A), with the best being about 100-fold better than the parent (*K*_D_ = 44 nM for the parent versus 0.46 nM for the mutant). Fig 2A and 2D show the sensorgrams for the parent bH1 and best triple mutant, respectively. We see a decreased k_off_ rate with respect to the parent. Table 1 lists the k_on_ and k_off_ rates for the parent sequence and triple mutants. For all the triple mutants the improvements in affinities were due primarily to slower k_off_ rates.

**Table 1:** SPR-measured k_on_ and k_off_ rates for the parent sequence and triple mutants.

Mutation						k_on_ (10^5^M^-1^s^-1^)	k_off_ (10^-4^s^-1^)	*K*_D_
**bH1-VEGF**								
Parent						2.2 ± 0.5	90 ± 17	44 ± 17 nM
H	D98M	H	Y33W	H	G99D	1.0 ± 0.1	1.4 ± 0.0	1.5 ± 0.2 nM
H	D98F	H	Y33W	H	G99D	1.4 ± 0.3	1.5 ± 0.0	1.1 ± 0.2 nM
H	D98W	H	Y33W	H	G99D	1.6 ± 0.3	0.7 ± 0.0	0.5 ± 0.1 nM
H	D98M	H	Y33W	H	G99M	2.1 ± 0.7	1.9 ± 0.0	1.0 ± 0.2 nM
H	D98F	H	Y33W	H	G99M	2.2 ± 0.6	2.0 ± 0.0	0.9 ± 0.2 nM
H	D98W	H	Y33W	H	G99M	2.9 ± 0.7	3.4 ± 0.2	1.2 ± 0.2 nM
**bH1-HER2**								
Parent						3.7 ± 0.4	13 ± 1.6	3.6 ± 0.6 nM
H	D98Y	H	D31K	L	I29K	22 ± 5	2.8 ± 1.1	0.14 ± 0.09 nM
H	D98F	H	D31K	L	I29K	22 ± 4	2.9 ± 0.6	0.14 ± 0.05 nM
H	D98K	H	D31K	L	I29K	23 ± 4	3.9 ± 1.0	0.15 ± 0.05 nM
H	D98Y	H	D31K	L	I29R	38 ± 4	2.5 ± 0.5	0.07 ± 0.01 nM
H	D98F	H	D31K	L	I29R	27 ± 5	2.9 ± 0.6	0.11 ± 0.02 nM
H	D98K	H	D31K	L	I29R	44 ± 6	4.5 ± 0.3	0.11 ± 0.02 nM
**Herceptin-HER2**								
Parent						11 ± 4	0.57 ± 0.09	58 ± 18 pM
H	D102F	H	D31K	L	D28K	61 ± 8	0.11 ± 0.00	1.9 ± 0.2 pM
H	D102F	H	D31M	L	D28K	51 ± 13	0.14 ± 0.03	2.9 ± 0.7 pM
H	D102F	H	D31K	L	D28R	68 ± 4	0.17 ± 0.07	2.5 ± 1.0 pM
H	D102F	H	D31M	L	D28R	50 ± 11	0.19 ± 0.04	4.0 ± 1.4 pM

**Fig 3:**
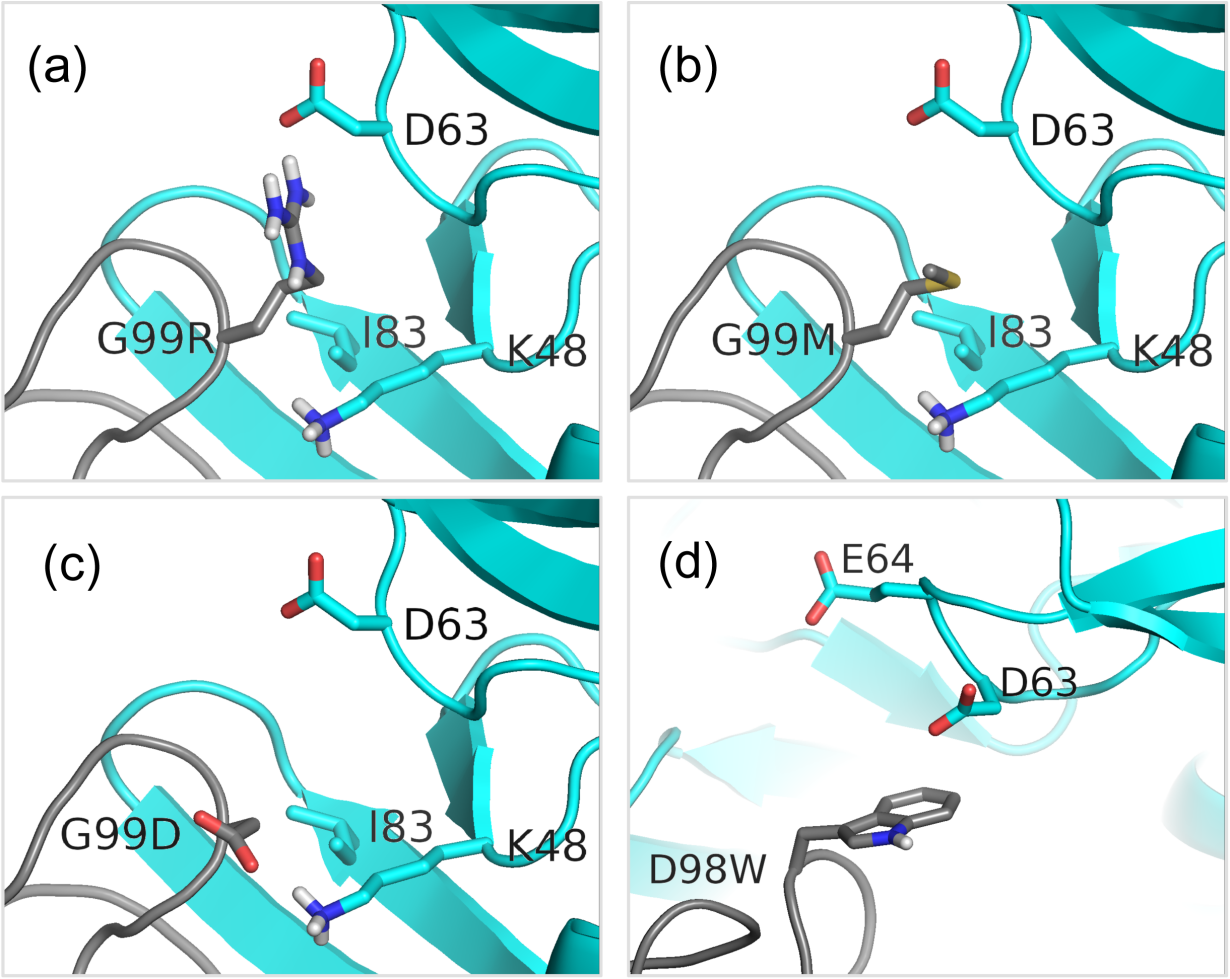
Structural basis for increased affinities of the HD98W and HG99X mutations for bH1-VEGF binding. The bH1 and VEGF chains are colored grey and cyan, respectively. (a) Modeled binding mode of the HG99R. A salt bridge is formed between HG99R of bH1 and D63 of VEGF. (b) Modeled binding mode of HG99M. Nonpolar interactions are formed between HG99M of bH1 and I83 and K48 of VEGF. (c) Modeled binding mode of the HG99D. A salt bridge is formed between HG99D of bH1 and K48 of VEGF. (d) HD98W removes unfavorable electrostatic interactions of HD98 with D63 and E64 of bH1.

Fig 3 illustrates the structural basis for some of these improvements in affinity. HD98 has unfavorable electrostatic interactions with two nearby carboxylates (D63 and E64) on VEGF (Fig 3D). Mutating it to Trp eliminates the bad electrostatics and introduces new van der Waals contacts. On the G99 site, mutation to Asp introduces a salt bridge with K48 on VEGF (Fig 3C). Interestingly, the oppositely charged mutation HG99R also results in improved affinity, albeit less than with HG99D. In this case, HG99R forms a salt bridge with D63 on VEGF (Fig 3A). Also, the methylene groups of the HG99R and VEGF K48 side chains form nonpolar interactions. Yet another way on improving on HG99 is the HG99M mutation. Instead of creating salt bridges it introduces nonpolar interactions between the Met side chain and side chains of VEGF I83 and K48 (Fig 3B). We see that even on the same site, there can be quite varied mechanisms of enhancing binding affinity. This also demonstrates the versatility of our method, which is capable of suggesting multiple strategies for improving mutations that do not rely solely on electrostatics. Earlier studies by Lippow et al. suggested that it was difficult to reliably predict affinity enhancements arising from mutations to larger amino acids and they relied more on electrostatics as a predictor of changes in binding free energy [4]. More recently, Kiyoshi et al. carried out an exhaustive virtual single-mutant scan of the antibody 11K2 against the chemokine MCP-1 and obtained single mutants that had up to 4.6 fold higher affinity than the wild type as measured by SPR [7]. All their successful mutants were also mutations to charged amino acids. Unlike this work, they did not go further and construct double and higher order mutants from their single mutations. In this context, the designed HY33W and HG99M mutants are informative as they do not alter the net charge and rely on an increase of the size of the side chain upon mutation to improve binding affinity.

A key aspect of our protocol is the experimental validation of designed single mutants prior to designing and producing double mutants. To illustrate the importance of this step consider the single mutants HD98M and LS30dM. The z-scores for each of these mutants were within the top 5 in the virtual single mutant scan (S1 Table). Thus, it would have been tempting to immediately make the double mutant. However, experimental validation of the single mutants showed that LS30dM is in fact destabilizing rather than enhancing, suggesting that making a double mutant incorporating LS30dM would have been a waste of time and resources. To confirm this, the double mutant with HD98M and LS30dM was made and resulted in a greater than 10-fold drop in binding affinity.

The second affinity maturation test was carried out on bH1-HER2. Fig 1B summarizes the results for three rounds of optimization. In the first round, four sites each on the heavy and light chains were identified as candidates for mutation. Thirteen single mutants were produced and tested, with z-scores ranging from –3.1 to –0.9 (S1 Table). Nine showed improvements in affinity, albeit somewhat more modest than what was observed for the bH1-VEGF case. In round two, 12 double mutants out of a possible 25 (S3 Table) involving four sites were made and tested, all of which showed further gains in affinity. In the third round, 6 triple-mutant combinations of the mutants in round two were made. All the triple mutants showed further increases in affinity, with the best achieving about a 50-fold improvement (parent *K*_D_ = 3.6 nM versus 0.066 nM for the mutant). Fig 2B and 2E show the sensorgrams for the parent bH1 and best triple mutant, respectively. The improvement in affinity arises from changes in both the k_on_ and k_off_ rates (Table 1). The increases in k_on_ contribute about a 6- to 10-fold improvement in *K*_D_ and about a 3- to 6-fold contribution from decreases in k_off_. The change in k_on_ is likely due to the long-range electrostatic interactions [10,11] between the new Lys and Arg residues in the mutants and the net negative charge of HER2.

The third affinity maturation test was carried out on Herceptin-HER2. We considered this a particularly challenging test for affinity maturation since Herceptin is already such a tight binder (*K*_D_ = 58 pM). Fig 1C summarizes the results for three rounds of optimization. In the first round, two heavy-chain sites and two light-chain sites were identified as candidates for mutation. Eleven single mutants (z-scores ranging from –3.8 to –1.0, S1 Table) were produced, nine of which showed some improvement in affinity. In the second round, seven double mutants were tested and all showed improvements over the single mutant components. The double mutants incorporating the heavy chain HD102F had the best affinity and were used to generate triple mutants with light chain single mutant LD28R and LD28K, respectively. Unfortunately, triple mutants incorporating the most active double mutant (HD102F, HD31R) were heterogeneous upon purification and so were not included in the binding affinity measurements. The best triple mutant of the remaining four had about a 30-fold better affinity than parent (mutant *K*_D_ = 1.9 pM). Fig 2C and 2F show the sensorgrams for the parent Herceptin and best triple mutant, respectively. The contributions to the change in affinity break down into approximately 6-fold and 5-fold changes in k_on_ and k_off,_ respectively (Table 1). The four triple mutants also exhibited improvements in apparent binding affinity relative to the parent Herceptin Fab when tested by flow cytometry on two cell lines expressing the HER2 antigen at different cell surface densities (S1 Fig, S5 Table). Hence, the SPR-based binding affinities to the HER2 ectodomain are qualitatively consistent with the trend observed for binding to the full-length HER2 expressed at the cell surface.

**Fig 4:**
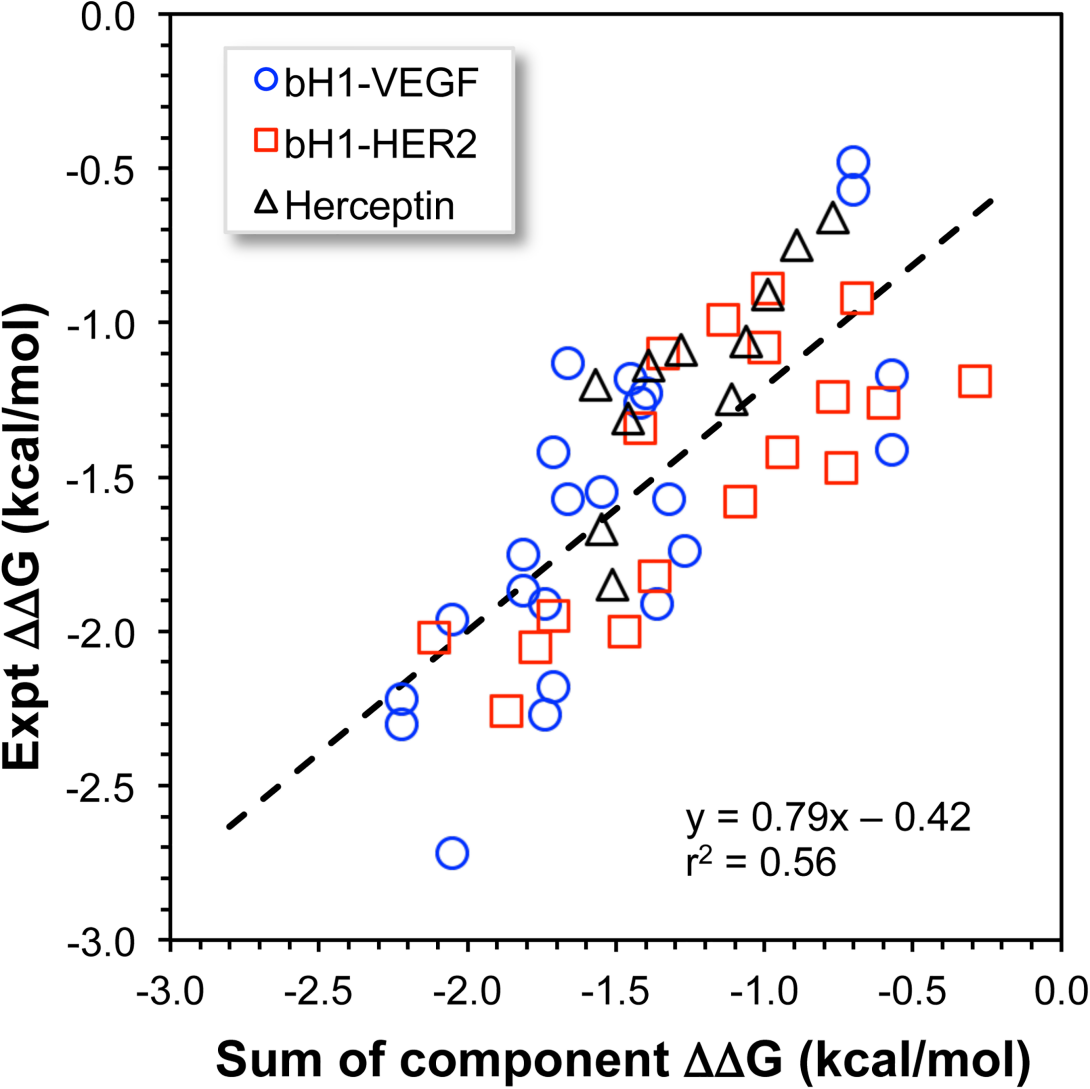
Additivity of contribution of mutations to binding affinity. Shown is a scatter plot of the experimentally measured relative binding affinities of double and triple mutants versus the sum of independently measured relative binding affinities of the component single/double mutants. The dashed line is the linear regression line for the entire set.

Implicit in our approach of stepwise build-up of mutations is the assumption of additivity in the contribution of each mutation to the binding free energy. The data generated in the multiple cycles of maturation allows us to examine this assumption. Fig 4 is a plot of the experimentally measured binding free energy contributions of double/triple mutants versus the sum of the contributions of their component single/double mutants. For the combined data set of all three systems we have a correlation of r^2^ = 0.56 and a slope of 0.79. If we force the intercept to be zero we obtain an r^2^ = 0.48 and a slope of 1.07. We see that the assumption of additivity is quite reasonable for this data set. One possible reason is that the component mutations have all been pre-selected to be favorable mutations. It is likely that the sum of favorable and unfavorable contributions would be less predictable.

## Discussion

The use of a consensus z-score is central to the ADAPT protocol. In a previous study [12] we examined the ability of consensus scores for predicting relative binding affinities in antibody-antigen complexes. We found that a consensus z-score using the scoring functions SIE, FOLDEF and Talaris-interface improved the transferability across different systems for enrichment predictions of mutants with improved binding. That study was a retrospective analysis of data in the SiPMAB database [12], which contains mutational, structural and binding affinity data for antibody-antigen complexes culled from the literature. However, a true test of the predictive ability of the consensus z-score is to apply it to the design of novel proteins with enhanced binding affinities. In this work, we used the consensus z-score approach for affinity maturation of three antibody-antigen complexes with initial K_D_ values ranging from a modest 44 nM to a tight 56 pM, achieving significant improvement in all three cases. Furthermore, the over 100 mutants across the three systems that were predicted to improve binding affinity were experimentally found to be highly enriched with enhanced binders. This suggests that the consensus z-score used has utility and transferability beyond the retrospective analysis of the SiPMAB database.

Further support of the conclusions of the previous study [12] about the value of using a consensus z-score is given below. Table 2 lists the z-scores and ranks for some selected single mutants for the three scoring functions used in this study–SIE, FOLDEF and Talaris-interface. For example, in the bH1-VEGF system the G99D mutation (a component of the best triple mutant) was identified only by SIE (z-score –6.0, rank 32). FoldEF gave a z-score of 0.56 and rank 522. Talaris-interface gave a z-score of 0.58 and rank of 725. The consensus z-score was –1.6, which brought it within the top 50 average z-scores. Using an average rank would have penalized the low ranks too much and not have given enough weight to the very favourable SIE score relative to the scores of the other single mutants. For the bH1-HER2 system, only FoldEF scored I29R highly (z-score –3.0, rank 19). The values for SIE and Talaris-interface were (z-score –0.88, rank 107) and (z-score 0.0, rank 429), respectively. The consensus z-score of –1.2 for I29R brought it into the top 50 average z-scores. For the herceptin-HER2 system, D102F was in the top 50 only for Talaris-interface (z-score –0.81, rank 27). The values for FOLDEF and SIE are (z-score –0.8, rank 88) and (z-score –1.5, rank 51), respectively. The consensus z-score was –1.0 bringing it within the top 50. The use of the consensus z-score allowed us to retain these single mutants as candidates for experimental validation. They eventually ended up as being part of the best triple mutants and would likely have been missed in the absence of the consensus z-score. Our results are consistent with our earlier findings that the use of consensus z-scores for ranking antibody-antigen binding affinities increases the robustness of the predictions [12].

**Table 2:** Individual scoring function z-scores and ranks for selected single mutants.

Mutant^a^	FoldEF		SIE		Talaris-interface		Consensus
	z-score	rank	z-score	rank	z-score	rank	z-score^c^
HG99D	0.6	522	–6.0	32	0.6	725	–1.6
LI29R	–3.0	19	–0.9	107	0.0	153^b^	–1.3
HD102F	–0.8	88	–1.5	51	–0.8	27	–1.0

^a^ HG99D, LI29R and HD102F are single mutants for bH1-VEGF, bH1-HER2 and herceptin-HER2, respectively. They are found in the best triple mutants for each of the systems.

^b^ This rank is shared with 404 other mutants with a z-score of 0.0.

^c^ The consensus z-score is the arithmetic average of the z-scores for the three scoring functions. All three consensus z-scores are within the top 50 for their respective systems. In contrast, the average of the ranks would not have placed the mutants within the top 50.

An important limitation of ADAPT is the need for a three-dimensional structure of the antibody-antigen complex. Ideally this would be from a crystal structure for the parent sequence or from a high-quality model based on a closely related structure. We expect the reliability of the scoring functions used will be quite sensitive to the accuracy of the structure used as a starting point for modeling the mutations. A related limitation of ADAPT is that it is a very conservative exploration of conformational space. Although we allow some limited movement and repacking of side chains in SIE_SCWRL and FOLDX, the backbone conformation is essentially assumed to remain unchanged by the various mutations. Of course, in general, backbone conformations can be sequence-dependent. However, the spirit of ADAPT is to optimize the amino acid sequence for a given backbone conformation. The expectation is that the side chains that are designed will be highly compatible with the given backbone in the context of the antibody-antigen complex. Another limitation of ADAPT in its present form is that we do not allow for insertions or deletions of amino acids. This certainly precludes whole collections of mutation possibilities. However, in the examples presented here we find that even when the CDR chain lengths are kept fixed the sequence space is rich enough to provide mutants yielding significant improvements in affinity.

The application of ADAPT to the three systems described here focused on improving affinity and did not explicitly take developability into account. A possible concern is that the presence of Trp and Met in some of our optimized sequences could introduce potential sites of oxidation and compromise the viability of the mutated antibody as a commercial product. In an actual drug discovery campaign, one could exclude Met and Trp in the mutation scans. In our case, exclusion of those amino acids would have resulted in the selection of other single mutants among the top 50. At this point we don’t know whether the resulting mutants would have produced comparable affinity enhancements for bH1 against VEGF. However, we note that the best bH1 and herceptin triple mutants against HER2 contain neither Trp nor Met. Hence, the platform is capable of achieving affinity enhancements without the introduction of Trp or Met mutations.

## Conclusions

We have presented ADAPT, a combined interleaved computational and experimental protocol and workflow for affinity maturation of antibodies. We summarize the main results below.

1. An exhaustive virtual scan of single mutants followed by experimental validation of selected single mutants avoids the combinatorial explosion in exploring multi-site mutations.
2. Validation at the single mutation stage improves the robustness and efficiency of designing double and triple mutations, leading to increased binding affinities in three test systems of about 100-, 50- and 30-fold, respectively. It took less than 40 mutants for each system to achieve these improvements. 100% of double and triple mutants showed affinity improvements versus their parent molecules. The success rate across all mutants was 90%.
3. Reasonable additivity of the contribution to binding free energy of mutations was observed.
4. Electrostatics is a major but not exclusive contributor to enhanced affinity. Examples of mutations to non-polar groups that improve affinity were also observed.
5. Enhanced binding of the designed mutants was achieved by increasing the binding on-rate, decreasing the off-rate or a combination of both. This can arise from a combination of long-range electrostatics with short-range non-polar interactions.
6. The data set of over 100 SPR binding affinity measurements in three congeneric series with mostly affinity-enhancing mutations will be of great value to the development, calibration and validation of computational tools for antibody design.

The tight interleaving of virtual mutations and experimental validation gives rise to the success of ADAPT, which promises to be a useful platform for affinity maturation of antibodies. While this strategy has been validated on affinity maturation one can envision expanding the applicability domain of ADAPT towards the optimization of other important biophysical properties along with other biotherapeutic frameworks.

## Methods

### Structure preparation

The initial structures of the bH1-VEGF, bH1-HER2 and Herceptin-HER2 complexes were based on the crystal structures 3bdy, 3be1 and 1n8z, respectively. Only the V_H_ and V_L_ regions of the Fab were used in the simulations. From the HER2 extracellular domain, all residues N-terminal to Cys489 were deleted to reduce the size of the antigen for the calculations. HER2 residues 581–590 are not visible in either 3be1 or 1n8z. The missing loop was reconstructed by grafting in the loop from the crystal structure 1n8y. Crystallographic water and counterions were removed. Hydrogen atoms were added and protonation states at neutral pH were adopted. Polar hydrogen orientations were visually examined and manually adjusted as needed. Structural refinement was then carried out by energy-minimization using the AMBER force-field [13–15], with a distance-dependent dielectric (4*r*_ij_) and infinite cutoff for non-bonded interactions. Non-hydrogen atoms were restrained at their crystallographic positions with a harmonic force constant of 5 kcal/(mol^.^A^2^).

### Building and scoring mutants

We used three protocols (SIE-SCWRL, FoldX and Rosetta) for modeling mutants and scoring their binding affinities. For each of these protocols, additional refinement of the AMBER-refined parent structure was carried out as prescribed by each of the methods as a starting point for building and scoring the mutants. We describe each of these methods briefly below. A more detailed description of the procedures can be found in Sulea et al.[12]

#### SIE-SCWRL

This is a hybrid method. SCWRL version 4.0 (Fox Chase Cancer Center, Philadelphia, PA; http://dunbrack.fccc.edu/scwrl4) [16] was used to build and repack the mutant side chains, keeping the rest of the complex fixed. The resulting SCWRL-generated structures of the mutants were then energy-minimized with the AMBER force-field [13–15], allowing only a set of residues 6 Å around the mutated residue to be mobile. The Fv-antigen interaction was scored with the SIE [17,18] function. SIE was originally developed for predicting binding affinities of small molecules with proteins. We use the scoring function here with no alteration of its default parameters.

#### FoldX

Two types of scores were calculated with FoldX version 3.0b6 (Center for Genomic Regulation, Barcelona, Spain; http://foldx.crg.es) [19,20], Fv-antigen binding affinity scores and Fv stability scores. The RepairPDB routine of FoldX was used to prepare a relaxed structure of the complex prior to mutation. Default parameters were used throughout along with setting the ionic strength to 0.1 M. FoldX was used to repack mutated side chains, keeping the backbone fixed. Side chains in the vicinity of the mutations were also repacked. Binding affinities were calculated using the FOLDEF [19,20] scoring function. The stability of the isolated mutant Fv relative to the parent was also estimated. Any mutant Fv that was predicted to be more than 100-fold less stable than the parent was excluded from consideration by all affinity scoring protocols. Since the repacking step is a stochastic process and produces non-identical results for each run, ten independent runs were carried out for each mutant and a Boltzmann-weighted average was taken.

#### Rosetta

We used Rosetta software version 3.5 (University of Washington, Seattle, WA; http://www.rosettacommons.org) [21,22] to repack and relax only the mutated side chain in a rigid protein environment. The default weights of the Talaris 2013 scoring function was used for the repacking and relaxation step, followed by rescoring of binding affinity with a modified function, Talaris-interface, whose weights were optimized for alanine scanning mutations (https://guybrush.ucsf.edu/benchmarks/benchmarks/alanine_scanning) [22].

#### Consensus Z-score

The z-score indicates how many standard deviations a particular value in a series of values is from the mean of all the values.

$$z_{i}=\frac{x_{i}-x_{\text{mean}}}{\sigma }$$

where σ is the standard deviation of the scores. It provides a convenient way of normalizing the results of various scoring functions and combining them in a useful way. The consensus z-scores reported in S1 Table are simply the average z-scores of the mutants across the three binding affinity scoring functions used: SIE, FoldX (FOLDEF) and Rosetta (Talaris-interface). The combination of these three scoring functions was found to give good Spearman rank-order correlations for binding affinity on an antibody-antigen data set of about 200 single-point mutants [12]. Consensus z-scores have been used previously in virtual screening of chemical libraries [23].

Some scoring function “energies” may be unusually large in magnitude (e.g., when steric clashes are present) and will skew the mean and standard deviations. To alleviate this we use a modified z-score instead that is based on the median and median absolute deviation [24]. The median absolute deviation (MAD) is more robust to outliers than the standard deviation. The modified z-score, *z*_i_, of a particular value *x*_i_ is defined as

$$z_{i}=\frac{x_{i}-x_{\text{med}}}{1.4826\times \text{MAD}}$$

where *x*_med_ is the median value. The multiplicative constant 1.4826, which converts the MAD to a standard deviation assuming a normal distribution, is obtained by noting that the MAD is the median of the half-normal distribution and hence can be calculated from the inverse error function

$$\text{MAD}=\sigma \sqrt{2}{\text{erf}}^{-1}(0.5)=0.6749\sigma$$

where σ is the standard deviation.

With the Rosetta Talaris-interface scoring function used in this study, most of the scores were zero or very close to zero, resulting in a MAD that was zero or very close to zero. This makes the z-score undefined or ill-behaved. In this case we used the mean absolute deviation (MeanAD) instead of the MAD. The MeanAD, which is larger than the MAD, is always nonzero unless all values are identical. The z-score is then calculated as

$$z_{i}=\frac{x_{i}-x_{\text{mean}}}{1.2533\times \text{MeanAD}}$$

The multiplicative constant 1.2533 for the MeanAD is obtained by noting that the MeanAD is the mean of the half-normal distribution.

$$\text{MeanAD}=\sigma \sqrt{2/\pi }$$

The ADAPT software was set to automatically switch to using MeanAD if MAD < 0.03.

### Selection of mutants

Lists of the single-point mutants corresponding to the top 50 consensus z-scores for each of the three complexes are shown in the S1 Table. A subset of the top 50 was selected for experimental validation. The reduction of the top-50 list to a smaller subset was driven primarily by cost considerations. In an actual drug discovery setting, it would not be unreasonable to test all 50 single mutants at this stage. The selection of the subset was based not solely on the values of the composite z-scores. A previous study showed that while a collection of mutants with favorable z-scores will be enriched in true positives, the z-scores are only modestly accurate in ranking binding affinities within the set [12]. Rather than choosing strictly by z-scores, we also sought to have diversity in the mutation sites explored and in the residue types introduced. In addition, visual inspection of the modeled structures was used to further evaluate the proposed mutants. For example, for herceptin the LS50Y mutation had a slightly more negative consensus z-score than the LS50F and LS50W mutations largely due to the very favorable score given by FoldX. Examination of the FoldX predicted structure for LS50Y showed that the score was based on a structure that disrupted an internal salt bridge between D82 and K105 in HER2. Being internal to HER2, this loss of a salt bridge was not reflected in the FoldX interaction energy. We thus opted to go with the LS50F and LS50W mutations for which the aforementioned salt bridge was preserved in the predicted structures.

The validated single mutants that exhibit improved binding affinities were then used to construct virtual double mutants that were then scored using the three scoring functions. The small number of virtual double mutants makes it difficult to interpret z-scores calculated using the double-mutant score distribution in a statistically meaningful way. Instead, we calculated z-scores using the MAD (or MeanAD, as the case may be) and median of the corresponding single-mutant scan for that scoring function. This allowed a direct comparison of the double-mutant z-scores with the single-mutant ones to see if the double mutants showed further predicted improvements in affinity. The consensus z-scores were calculated as before, i.e., as arithmetic averages of individual z-scores. The double-mutant z-scores were primarily used to weed out any possible incompatible combinations of single mutants. For example, it is conceivable that two bulky mutations on adjacent sites may not be simultaneously viable. With the single mutants obtained in this work, such incompatibilities were not observed. In selecting the subset of double mutants for experimental validation we generally chose double mutants whose single-mutant components showed the best binding affinities experimentally. For example, for the bH1-VEGF system, we restricted the choice of double mutants involving HD98 to use only HD98M, HD98F or HD98W –the best single mutants at that site. Again, as in the single-mutant stage, it would be feasible in a drug discovery setting to skip the subset selection and test the entire double-mutant list.

The validated double and single mutants were then used to generate the triple mutants. S2–S4 Tables list the consensus z-scores for the double and triple mutants. The general workflow is shown schematically in S2 Fig.

### Calculation of K^WT^/K

Each batch of SPR measurements of mutant Fabs always included a determination of *K*^WT^ for the parent Fab as well. It is this measured *K*^WT^ for the specific batch that was used in calculating the *K*^WT^/*K* ratios (fold improvements in binding affinity) for the mutants. These ratios were averaged over replicate (three or more) measurements to provide the values tabulated in Fig 1. We felt that using a reference measured at the same time as the mutant would help reduce the effect of systematic errors and yield less noisy values for the relative improvements in affinity. In Fig 1 we also report the parent dissociation constant, $K_{\text{D}}^{\text{WT}}$, which is the overall average *K*^WT^ using multiple batches of SPR measurements across different mutants. One could use this overall average *K*^WT^ in calculating *K*^WT^/*K*, leading to slightly different numbers. For example, the improvements for the best triple mutants reported in Fig 1 are 104, 46 and 32, respectively for bH1-VEGF, bH1-HER2 and Herceptin-HER2 using our chosen approach. If we use the overall average *K*^WT^ instead, we obtain 96, 55 and 31 as the corresponding improvements. There is not a large discrepancy between the two approaches and we opted for the former one over the latter.

#### Fab production

cDNA for the designed Fab heavy and light chains were ordered from commercial vendors (GeneArt, Life Technologies). These contained signal peptide sequences and heavy-chain C-terminal His_8_ tags for purification. The Fabs were produced by co-transfection of CHO-3E7 cells. 2 x 10^6^ CHO-3E7 cells were plated in 2 ml of F17 medium. The cells were transfected with 2 μg total DNA (containing 500 ng each of the heavy chain and light chain constructs) using PEI. The cells were maintained at 37^°^C for 24 h after which they were transferred to 32^°^C for 6 days. The cell-culture supernatant was harvested and analyzed by SDS-PAGE for expression. His-tag purification was carried out and followed by UPLC-SEC, for verification of homogeneity as required.

### SPR measurements

GLC sensorchips, the Biorad ProteOn amine coupling kit (EDC, sNHS and ethanolamine), and 10mM sodium acetate buffers were purchased from Bio-Rad Laboratories Ltd. (Mississauga, ON). Recombinant soluble human HER2 extracellular domain (termed HER2 here) was purchased from eBioscience (San Diego, CA). Full-length isoform 165 of human VEGF-A (termed VEGF here) was produced recombinantly and purified. PBS running buffer with 0.05% Tween20 (PBST) was purchased from Teknova, Inc. (Hollister, CA).

All surface plasmon resonance assays were carried out using a BioRad ProteOn XPR36 instrument (Bio-Rad Laboratories Ltd., Mississauga, ON) at a temperature of 25C using PBS running buffer containing 0.05% Tween 20 (Teknova, Hollister, CA) with the addition of 3.4 mM EDTA. Three GLC chip channels were prepared for antigen immobilization by injecting a 1:10 dilution of the standard BioRad sNHS/EDC solutions for 140 s at 100 μL/min in the ligand direction. The first channel was used as a blank control; the remaining two channels were used for HER2 and VEGF immobilization. Immediately after the activation, 2.5 ug/mL solutions of HER2 and VEGF in 10 mM NaOAc pH 4.5 were injected in the ligand (vertical) direction at a flow rate of 25 μL/min until approximately 150 resonance units (RUs) were immobilized. Remaining active groups in all three channels were quenched by a 140 s injection of 1M ethanolamine at 100 μL/min in the ligand direction.

After immobilization of the antigens, each Fab variant was injected in the analyte (horizontal) direction for binding kinetics determination to the HER2 and VEGF surfaces. In each injection cycle, two buffer injections for 30 s at 100 μL/min in the ligand direction were used to stabilize the baseline. Next, 3-fold serial dilutions of each Fab variant (top nominal concentrations of 30, 60 or 120 nM) and a buffer blank were simultaneously injected over the blank, HER2 and VEGF surfaces at 50 μL/min for 120 s with a 900 s (or longer as required) dissociation phase. The SPR surfaces were regenerated by two 18-second pulses of 0.85% phosphoric acid at 100 μL/min to prepare for the next injection cycle. Sensorgrams were aligned and double-referenced using the buffer blank injection and the blank interspots, and the resulting sensorgrams were analyzed using ProteOn Manager software v3.1. The double-referenced sensorgrams were fit to the 1:1 binding model to determine the k_on_ (s^-1^M^-1^) and k_off_ (s^-1^). The binding affinity (*K*_D_) was determined from the ratio of k_off_/k_on_. Each Fab variant was injected in triplicate.

*Abbreviations*. EDC: *1-*ethyl*-3-(3-*dimethylaminopropyl*)* carbodiimide hydrochloride; sNHS: *N*-hydroxysulfosuccinimide; SPR: surface plasmon resonance; EDTA: ethylenediaminetetraacetic acid; PBS: phosphate buffered saline; HER2: Receptor-tyrosine kinase ErbB2 soluble ectodomain; VEGF165: 165 amino acid isoform of vascular endothelial growth factor.

### Cell-binding assay

#### Cell lines

The human breast cancer cell line MCF-7 (HTB-22) and the human ovarian SKOV-3 cells (HTB-77) were obtained from American Type Culture Collection (ATCC; Manassas, VA, USA) and maintained in recommended culture conditions. The cell line identities were authenticated with short tandem repeat (STR) profiling. Absence of mycoplasma contamination was tested by PCR.

#### Binding affinity measurement by flow cytometry analysis

Following treatment with cell dissociation solution (Sigma), cells were collected, washed and incubated for 2 hours on ice with Fabs at final concentrations ranging from 0.1 to 300 nM. A standard IgG isotype (human IgG; Jackson Immunochemicals) was used as negative control. Cells were then washed twice and incubated for 1 h on ice with 1/100 dilution of conjugated secondary antibody AF488-anti-human IgG (H+L) (Jackson Immunochemicals). Cells were washed and resuspended in medium containing 1% propidium iodide (Invitrogen). Each sample was filtered through a Nitex membrane and analyzed on a BD™LSRII flow cytometer (Beckton-Dickinson). Two thousand alive/single-cell events are acquired per sample. Fluorescence was evaluated using the BD FACSdiva™ Software.

#### Data analysis

Specific mean fluorescence intensity (MFI) is calculated for each sample point by subtracting the MFI value of negative control (background). Binding curves (specific MFI vs. linear or log antibody concentration) were fitted with GraphPad Prism 6 software using a One-site specific with Hill slope four-parameter nonlinear regression curve fitting model to determine apparent *K*_D_ values for each Fab.

## Supporting information

S1 Table Top 50 consensus Z-scores for single mutants. The first column in S1 Table lists the mutation sites involved in the top 50 consensus z-scores. The first letter (H or L) refers to the heavy or light chain, respectively. Scores in red correspond to mutants that were selected for production and experimental validation.
(PDF)

S2 Table Consensus z-scores for double and triple mutants of bH1-VEGF. The z-scores shown are relative to the distribution of scores in the exhaustive scan of single mutants, i.e., the median (or mean) absolute deviations of the single-mutant scores were used in computing the z-scores. In red are the mutants that were produced and validated experimentally. Z-scores were calculated for all double mutants arising from combinations of validated single mutants that showed an improvement in affinity (Fig 1, main text). For example, for bH1-VEGF experimental validation of double mutants involving the D98 site was restricted to those containing HD98M, HD98F or HD98W because those were found to be the best single mutants for that site (Fig 1, main text). The first letter (H or L) refers to the heavy or light chain, respectively. Scores in red correspond to mutants that were selected for production and experimental validation. The selection of double mutants to test took into account the improvement in affinity afforded by the single mutants together with the calculated z-score for the double-mutant combination.
(PDF)

S3 Table Consensus z-scores for double and triple mutants of bH1-HER2. The z-scores shown are relative to the distribution of scores in the exhaustive scan of single mutants, i.e., the median (or mean) absolute deviations of the single-mutant scores were used in computing the z-scores. In red are the mutants that were produced and validated experimentally.
(PDF)

S4 Table Consensus z-scores for double and triple mutants of Herceptin-HER2. The z-scores shown are relative to the distribution of scores in the exhaustive scan of single mutants, i.e., the median (or mean) absolute deviations of the single-mutant scores were used in computing the z-scores. In red are the mutants that were produced and validated experimentally. No double mutants involving light chain mutants LD28R or LD28K were made since the plan was to incorporate both of these in triple mutants anyway. In selecting which triple mutants to make, we decided to focus on the heavy chain double mutants with the best affinities as measured by SPR (Fig 1, main text).
(PDF)

S5 Table Apparent *K*_D_ values of Herceptin Fab and its triple mutants by flow cytometry analysis (n = 3). (PDF)

S1 Fig Binding titration curves to determine dissociation constants, *K*_D_. Mean fluorescence intensity (MFI) from FACS data is plotted versus the concentration of different anti-HER2 Fabs on MCF-7 (A) or SKOV-3 (B) cell lines. Data are fit using One-site specific with Hill slope four-parameter nonlinear regression curve fitting model (see Methods in main text). Independent triplicate titrations were performed on different days, with similar results. The parent Herceptin Fab is indicated with blue filled circles and blue curve, and its triple mutants from S5 Table with the other colors and symbols.
Panels A and B of **S1 Fig** show dose-response binding curves to MCF-7 and SKOV-3 cells for the Herceptin Fab (blue symbols) and its four affinity-matured triple mutants listed in **Fig 1C**. The data indicate that the affinity-matured variants have similar apparent binding among themselves and improved binding relative to the parental Herceptin Fab. This ranking is in qualitative agreement with the SPR data (see **Fig 1C** and **Table 1**). Apparent *K*_D_ values based on this cell-based assay are listed in **S5 Table**. One can note the smaller magnitudes of binding affinity improvements obtained with the cell-based experiments relative to the improvements obtained with the SPR assay, a consequence of the washing steps used in the cellular assay.
(PDF)

S2 Fig Affinity maturation workflow. (PDF)

S1 Appendix Fold improvements in binding affinity and relative changes in binding free energy (kcal/mol) relative to the parent Fab during three rounds of mutations (Excel spreadsheet of data in Fig 1). (XLSX)
