# Supplementary material for: Assisted Design of Antibody and Protein Therapeutics (ADAPT)
Source: PLoS One. 2017 Jul 27;12(7):e0181490. doi: 10.1371/journal.pone.0181490 (PMC5531539; doi:10.1371/journal.pone.0181490)
Supplement: S1 Table — The first column in S1 Table lists the mutation sites involved in the top 50 consensus z-scores. The first letter (H or L) refers to the heavy or light chain, respectively. Scores in red correspond to mutants that were selected for production and experimental validation. (PDF) [file pone.0181490.s001.pdf]

S1 Table. Top 50 consensus Z-scores for single mutants.

bH1-VEGF, WT Z-score = -0.19

	Res	R	K	Q	N	S	T	H	W	Y	F	M	L	I	V	A	G	E	D
H	D98	-5.4	-4.1	-3.8	-4.8	-3.5	-3.9	-4.3	-8.5	-6.4	-6.8	-6.5	-4.1	-3.5	-3.8	-3.2	-2.9		
H	G99	-7.6		-4.9				-3.5			-1.6	-2.3							-1.6
L	S30d	-3.0	-3.0	-2.3	-1.8		-3.3					-6.9	-6.1	-4.2	-2.5				
L	S30b	-6.3	-4.5					-2.3				-2.0							
L	T93			-1.9														-2.7	
H	Y52	-2.4							-2.2										
H	Y33	-1.6							-2.4			-1.8							
H	D31	-2.1	-2.4	-1.5		-1.5											-1.6		
L	Y53								-2.1										
H	T53	-1.8	-1.6																

bH1-HER2, WT Z-score = -0.21

	Res	R	K	Q	N	S	T	H	W	Y	F	M	L	I	V	A	G	E	D
H	D31	-2.0	-3.1	-1.8	-0.9			-1.3		-1.0		-1.3	-1.5	-1.2		-0.9			
H	D98	-1.2	-1.7	-1.3		-1.3	-1.6	-1.3	-2.1	-2.8	-2.7	-2.0	-2.3	-1.7	-1.1			-1.1	
L	G31	-2.1	-2.0	-1.2	-1.4	-1.2	-1.3	-1.6	-2.6	-2.2	-2.5	-2.4							
H	Y33	-2.0																	
H	Y52	-1.8																	
L	S56								-1.8			-1.0							
H	N54	-1.6	-1.1																
L	S30b			-1.5															
L	I29	-1.3	-0.9																
H	F100								-1.3										
L	S30d	-1.2																	
H	R58		-1.1																
L	T93			-1.0															
H	G99															-0.9			
H	Y56								-0.9										

Herceptin-HER2, WT Z-score = -0.16

	Res	R	K	Q	N	S	T	H	W	Y	F	M	L	I	V	A	G	E	D
H	D31	-3.8	-2.5	-1.4	-1.5	-1.2	-1.3	-1.3	-1.9	-1.9	-1.8	-1.6							
L	D28	-3.1	-1.9	-2.1	-1.5	-1.1	-1.0	-2.4		-0.9			-1.0	-1.0	-1.0	-1.1	-1.2		
L	S50				-1.1			-1.6	-2.2	-2.4	-2.0	-2.0	-1.3						
L	T31							-2.1											
H	D102	-1.1	-1.1					-1.8	-1.0		-1.0	-1.3	-1.0						
L	S56								-1.1	-1.0	-1.0	-1.3	-1.0	-1.3					
L	T93								-1.3										
L	Y92								-1.3										
H	T32											-1.1							
H	F104								-0.9				-0.9						

The first column in Table S1 lists the mutation sites involved in the top 50 consensus z-scores. The first letter (H or L) refers to the heavy or light chain, respectively. Scores in red correspond to mutants that were selected for production and experimental validation.
