# Supplementary material for: Assisted Design of Antibody and Protein Therapeutics (ADAPT)
Source: PLoS One. 2017 Jul 27;12(7):e0181490. doi: 10.1371/journal.pone.0181490 (PMC5531539; doi:10.1371/journal.pone.0181490)
Supplement: S2 Table — The z-scores shown are relative to the distribution of scores in the exhaustive scan of single mutants, i.e., the median (or mean) absolute deviations of the single-mutant scores were used in computing the z-scores. In red are the mutants that were produced and validated experimentally. Z-scores were calculated for all double mutants arising from combinations of validated single mutants that showed an improvement in affinity (Fig 1, main text). For example, for bH1-VEGF experimental validation of double mutants involving the D98 site was restricted to those containing HD98M, HD98F or HD98W because those were found to be the best single mutants for that site (Fig 1, main text). The first letter (H or L) refers to the heavy or light chain, respectively. Scores in red correspond to mutants that were selected for production and experimental validation. The selection of double mutants to test took into account the improvement in affinity afforded by the single mutants together with the calculated z-score for the double-mutant combination. (PDF) [file pone.0181490.s002.pdf]

S2 Table. Consensus z-scores for double and triple mutants of bH1-VEGF.

bH1 – VEGF							
**Double mutants**		**z-score**	**Triple mutants**			**z-score**
H D98W	L S30bR	-14.4	H D98W	H Y33W	H G99D	-12.8
H G99R	L S30bR	-13.7	H D98W	H Y33W	H G99M	-11.9
H D98F	L S30bR	-12.9	H D98M	H Y33W	H G99M	-11.1
H D98M	L S30bR	-12.7	H D98M	H Y33W	H G99D	-10.3
H D98Y	L S30bR	-12.5	H D98F	H Y33W	H G99D	-9.7
H D98W	H G99D	-12.1	H D98F	H Y33W	H G99M	-8.0
H D98W	H G99M	-11.8				
H D98M	H G99R	-11.2				
H D98F	H G99R	-11.0				
H D98H	H G99R	-10.8				
H D98Y	H G99D	-10.7				
H D98W	H G99R	-10.5				
H G99R	H Y33W	-10.4				
H D98H	L S30bR	-10.3				
H D98M	H G99M	-9.8				
H D98M	H G99D	-9.2				
H D98Y	H G99M	-9.1				
H D98F	H G99D	-9.1				
H D98W	H Y33W	-9.0				
H D98H	H G99M	-8.7				
H Y33W	L S30bR	-8.6				
H D98F	H G99M	-8.4				
H G99M	L S30bR	-8.2				
H D98M	H Y33W	-7.5				
H G99D	L S30bR	-7.5				
H D98H	H G99D	-6.8				
H D98Y	H G99R	-6.5				
H D98Y	H Y33W	-5.9				
H D98F	H Y33W	-5.7				
H D98H	H Y33W	-4.9				
H G99M	H Y33W	-4.4				
H G99D	H Y33W	-4.3				

The z-scores shown are relative to the distribution of scores in the exhaustive scan of single mutants, i.e., the median (or mean) absolute deviations of the single-mutant scores were used in computing the z-scores. In red are the mutants that were produced and validated experimentally. Z-scores were calculated for all double mutants arising from combinations of validated single mutants that showed an improvement in affinity (Figure 1, main text). For example, for bH1-VEGF experimental validation of double mutants involving the D98 site was restricted to those containing HD98M, HD98F or HD98W because those were found to be the best single mutants for that site (Figure 1, main text). The first letter (H or L) refers to the heavy or light chain, respectively. Scores in red correspond to mutants that were selected for production and experimental validation. The selection of double mutants to test took into account the improvement in affinity afforded by the single mutants together with the calculated z-score for the double-mutant combination.
