# Supplementary material for: Assisted Design of Antibody and Protein Therapeutics (ADAPT)
Source: PLoS One. 2017 Jul 27;12(7):e0181490. doi: 10.1371/journal.pone.0181490 (PMC5531539; doi:10.1371/journal.pone.0181490)
Supplement: S3 Table — The z-scores shown are relative to the distribution of scores in the exhaustive scan of single mutants, i.e., the median (or mean) absolute deviations of the single-mutant scores were used in computing the z-scores. In red are the mutants that were produced and validated experimentally. (PDF) [file pone.0181490.s003.pdf]

S3 Table. Consensus z-scores for double and triple mutants of bH1-HER2.

bH1 – HER2						
Double mutants		z-score	Triple mutants			z-score
H D31K	H D98F	-5.3	H D31K	H D98Y	L I29R	-6.7
H D31K	H D98Y	-5.3	H D31K	H D98F	L I29R	-6.7
H D31K	H Y52R	-4.8	H D31K	H D98Y	L I29K	-6.3
H D31K	H D98M	-4.7	H D31K	H D98F	L I29K	-6.3
H D31K	H D98W	-4.6	H D31K	H D98K	L I29R	-5.4
H D31K	L I29R	-4.3	H D31K	H D98K	L I29K	-5.2
H D31K	H D98K	-4.2				
H D98Y	L I29R	-4.0				
H D98F	L I29R	-4.0				
H D31K	L I29K	-3.9				
H D98W	L I29R	-3.6				
H D98Y	L I29K	-3.6				
H D98F	L I29K	-3.5				
H D98M	L I29R	-3.3				
H D98F	H Y52R	-3.3				
H D98Y	H Y52R	-3.2				
H D98W	L I29K	-3.2				
H D98W	H Y52R	-3.0				
H Y52R	L I29R	-3.0				
H D98M	L I29K	-2.9				
H D98K	L I29R	-2.8				
H Y52R	L I29K	-2.7				
H D98K	L I29K	-2.5				
H D98M	H Y52R	-2.4				
H D98K	H Y52R	-2.1				

The z-scores shown are relative to the distribution of scores in the exhaustive scan of single mutants, i.e., the median (or mean) absolute deviations of the single-mutant scores were used in computing the z-scores. In red are the mutants that were produced and validated experimentally.
