# Supplementary material for: Assisted Design of Antibody and Protein Therapeutics (ADAPT)
Source: PLoS One. 2017 Jul 27;12(7):e0181490. doi: 10.1371/journal.pone.0181490 (PMC5531539; doi:10.1371/journal.pone.0181490)
Supplement: S4 Table — The z-scores shown are relative to the distribution of scores in the exhaustive scan of single mutants, i.e., the median (or mean) absolute deviations of the single-mutant scores were used in computing the z-scores. In red are the mutants that were produced and validated experimentally. No double mutants involving light chain mutants LD28R or LD28K were made since the plan was to incorporate both of these in triple mutants anyway. In selecting which triple mutants to make, we decided to focus on the heavy chain double mutants with the best affinities as measured by SPR (Fig 1, main text). (PDF) [file pone.0181490.s004.pdf]

S4 Table. Consensus z-scores for double and triple mutants of Herceptin-HER2.

Herceptin – HER2						
Double mutants		z-score	Triple mutants			z-score
H D31R	L D28R	-6.8	H D102H	H D31R	L D28R	-6.5
H D31K	L D28R	-5.5	H D102M	H D31R	L D28R	-6.4
H D31R	L D28K	-5.4	H D102M	H D31K	L D28R	-6.2
H D102H	L D28R	-4.9	H D102H	H D31K	L D28R	-6.1
H D31M	L D28R	-4.7	H D102F	H D31K	L D28R	-5.5
H D102M	L D28R	-4.4	H D102F	H D31R	L D28R	-5.3
H D102K	L D28R	-4.3	H D102H	H D31R	L D28K	-5.2
H D31K	L D28K	-4.1	H D102M	H D31K	L D28K	-5.0
H D102F	L D28R	-4.0	H D102M	H D31R	L D28K	-4.9
H D102H	L D28K	-3.7	H D102F	H D31M	L D28R	-4.9
H D31M	L D28K	-3.6	H D102H	H D31K	L D28K	-4.8
H D102H	H D31R	-3.3	H D102F	H D31K	L D28K	-4.4
H D102M	H D31R	-3.3	H D102F	H D31R	L D28K	-4.2
H D102H	H D31K	-3.2	H D102F	H D31M	L D28K	-3.8
H D102M	L D28K	-3.1				
H D102M	H D31K	-3.1				
H D102K	L D28K	-3.0				
H D102K	H D31K	-2.8				
H D102F	L D28K	-2.7				
H D102F	H D31K	-2.6				
H D102M	H D31M	-2.4				
H D102F	H D31R	-2.4				
H D102K	H D31R	-2.2				
H D102F	H D31M	-1.9				
H D102H	H D31M	-1.7				
H D102K	H D31M	-1.6				

The z-scores shown are relative to the distribution of scores in the exhaustive scan of single mutants, i.e., the median (or mean) absolute deviations of the single-mutant scores were used in computing the z-scores. In red are the mutants that were produced and validated experimentally. No double mutants involving light chain mutants LD28R or LD28K were made since the plan was to incorporate both of these in triple mutants anyway. In selecting which triple mutants to make, we decided to focus on the heavy chain double mutants with the best affinities as measured by SPR (Figure 1, main text).
