# Supplementary material for: Assisted Design of Antibody and Protein Therapeutics (ADAPT)
Source: PLoS One. 2017 Jul 27;12(7):e0181490. doi: 10.1371/journal.pone.0181490 (PMC5531539; doi:10.1371/journal.pone.0181490)
Supplement: S5 Table — (PDF) [file pone.0181490.s005.pdf]

S5 Table. Apparent K_D values of Herceptin Fab and its triple mutants by flow cytometry analysis (n=3).

Fab variant	Apparent K_D (nM)	
	SKOV3 cells	MCF-7 cells
Herceptin parent	9.0 ± 2.2	2.9 ± 0.3
HD102F HD31M HD28K	4.6 ± 1.0	1.3 ± 0.1
HD102F HD31K HD28K	4.5 ± 0.7	1.1 ± 0.05
HD102F HD31K HD28R	5.4 ± 1.4	1.1 ± 0.1
HD102F HD31M HD28R	5.2 ± 1.7	1.1 ± 0.04
