# Supplementary material for: Assisted Design of Antibody and Protein Therapeutics (ADAPT)
Source: PLoS One. 2017 Jul 27;12(7):e0181490. doi: 10.1371/journal.pone.0181490 (PMC5531539; doi:10.1371/journal.pone.0181490)
Supplement: S1 Fig — Mean fluorescence intensity (MFI) from FACS data is plotted versus the concentration of different anti-HER2 Fabs on MCF-7 (A) or SKOV-3 (B) cell lines. Data are fit using One-site specific with Hill slope four-parameter nonlinear regression curve fitting model (see Methods in main text). Independent triplicate titrations were performed on different days, with similar results. The parent Herceptin Fab is indicated with blue filled circles and blue curve, and its triple mutants from S5 Table with the other colors and symbols. Panels A and B of S1 Fig show dose-response binding curves to MCF-7 and SKOV-3 cells for the Herceptin Fab (blue symbols) and its four affinity-matured triple mutants listed in Fig 1C. The data indicate that the affinity-matured variants have similar apparent binding among themselves and improved binding relative to the parental Herceptin Fab. This ranking is in qualitative agreement with the SPR data (see Fig 1C and Table 1). Apparent KD values based on this cell-based assay are listed in S5 Table. One can note the smaller magnitudes of binding affinity improvements obtained with the cell-based experiments relative to the improvements obtained with the SPR assay, a consequence of the washing steps used in the cellular assay. (PDF) [file pone.0181490.s006.pdf]

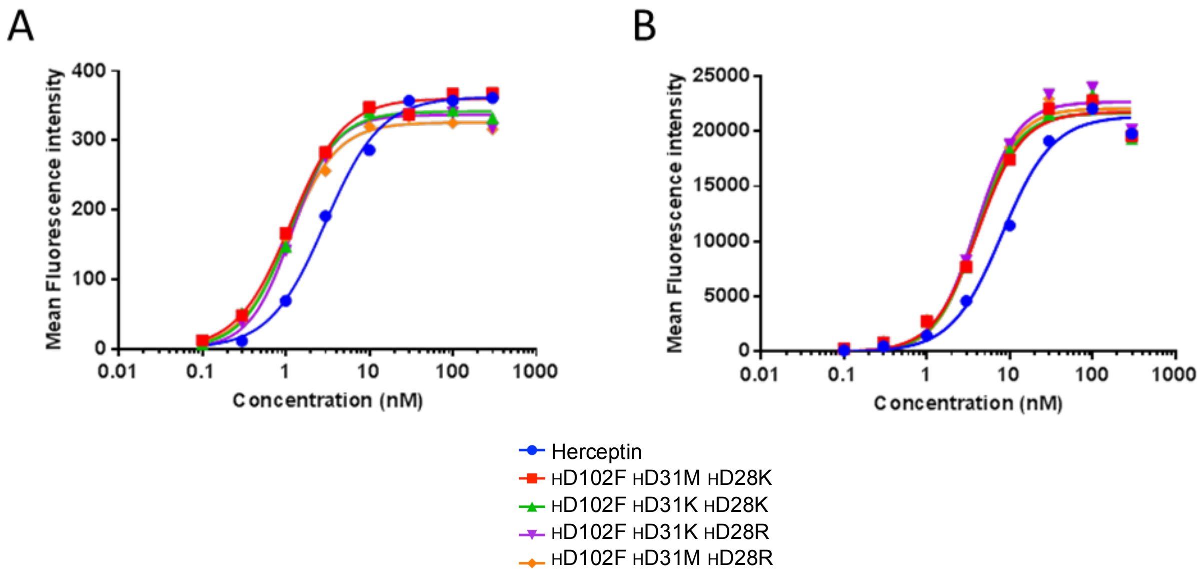

S1 Fig. Binding titration curves to determine dissociation constants, K_D. Mean fluorescence intensity (MFI) from FACS data is plotted versus the concentration of different anti-HER2 Fabs on MCF-7 (A) or SKOV-3 (B) cell lines. Data are fit using One-site specific with Hill slope four-parameter nonlinear regression curve fitting model (see Methods in main text). Independent triplicate titrations were performed on different days, with similar results. The parent Herceptin Fab is indicated with blue filled circles and blue curve, and its triple mutants from Table S6 with the other colors and symbols.

Panels A and B of **S1 Fig** show dose-response binding curves to MCF-7 and SKOV-3 cells for the Herceptin Fab (blue symbols) and its four affinity-matured triple mutants listed in **Figure 1C**. The data indicate that the affinity-matured variants have similar apparent binding among themselves and improved binding relative to the parental Herceptin Fab. This ranking is in qualitative agreement with the SPR data (see **Figure 1C** and **Table 1**). Apparent K_D values based on this cell-based assay are listed in **S5 Table**. One can note the smaller magnitudes of binding affinity improvements obtained with the cell-based experiments relative to the improvements obtained with the SPR assay, a consequence of the washing steps used in the cellular assay.
