# Supplementary figures and images for: Assisted Design of Antibody and Protein Therapeutics (ADAPT)
Source: PLoS One. 2017 Jul 27;12(7):e0181490. doi: 10.1371/journal.pone.0181490 (PMC5531539; doi:10.1371/journal.pone.0181490)

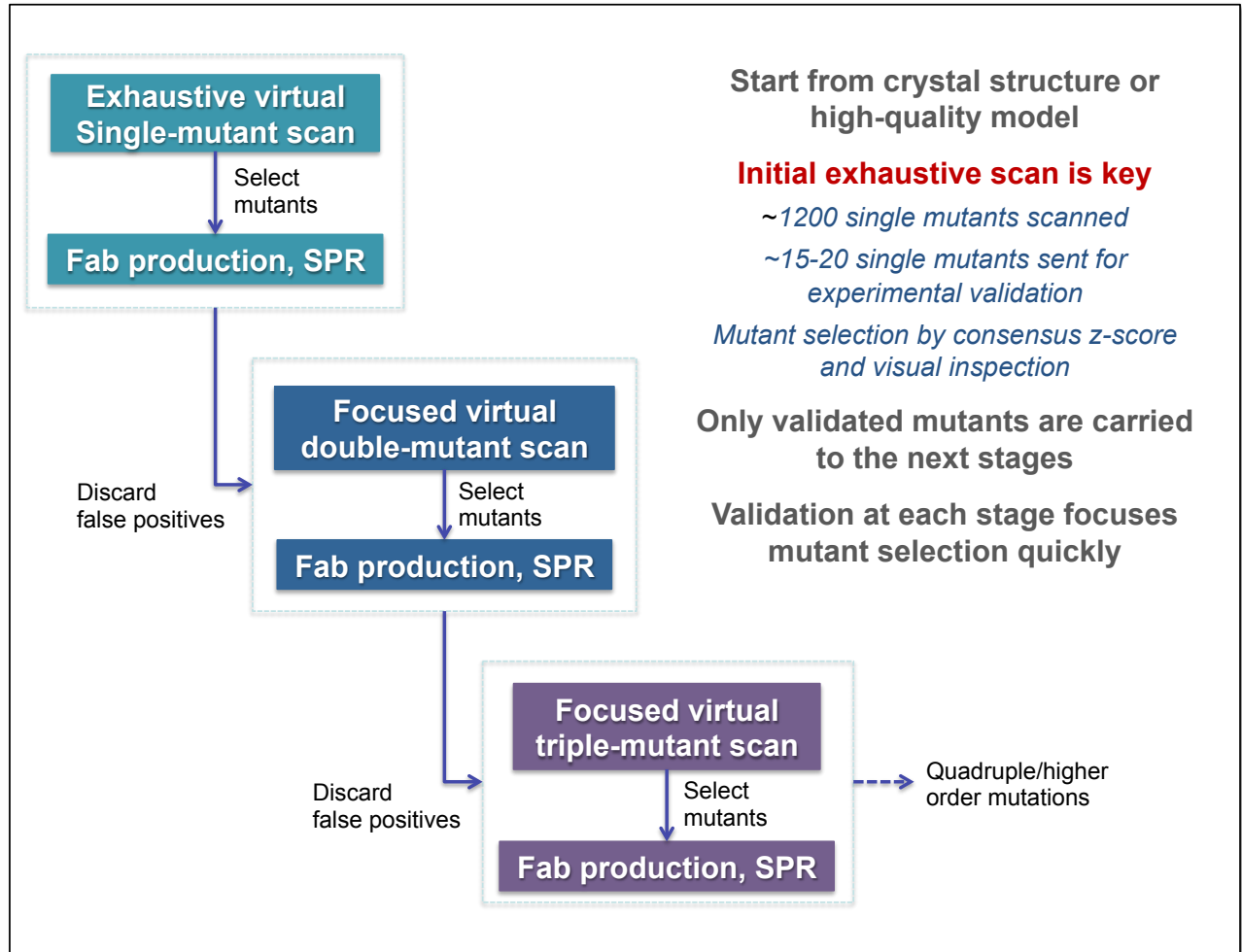

**S2 Fig.** Affinity maturation workflow.

Supplement: S2 Fig — (PDF) [file pone.0181490.s007.pdf]
